# How the Level of Noise Affects Temporal Accuracy of a QRS Detector—Case Study

**DOI:** 10.3390/s26010015

**Published:** 2025-12-19

**Authors:** Wojciech Reklewski, Piotr Augustyniak

**Affiliations:** Faculty of Electrical Engineering, Automatics, Computer Science and Biomedical Engineering, AGH University of Krakow, 30-059 Krakow, Poland

**Keywords:** QRS detector, electrocardiogram, detection noise immunity, detection jitter

## Abstract

Background: QRS complex detection is a key processing step of automated ECG analysis and determines its overall quality. The purpose of this paper is to study the detection performance of probably the most frequently implemented ready-to-use QRS detector in the presence of noise and with tightened temporal tolerance of detection points. Methods: We applied commonly used detection statistics (Detection Error Rate, Sensitivity, Positive Predictive Value, and *F*_1_ score), but re-defined true positive detection based on variable time jitter between detected and reference points. We also applied a controlled level of mixed noise to assess the detector’s performance in true-to-life conditions. Results: We found the following: (1) the detector under test showed a considerable drop in quality when reducing the jitter between 97.23 ms (*DER* = 8.08%) and 86.12 ms (*DER* = 67.22%), which means that the detection points’ time series are not accurate enough to be directly used for ECG time analysis; (2) with jitter allowed to 163.90 ms and an increasing noise level (*SNR* from 20 dB to −7.96 dB), the detection quality drops (*DER* from 0.98% to 57.13% respectively); however, an analysis of individual files revealed records, where the algorithm performs better in the presence of noise; (3) with a step-by-step code execution analysis of ECG strips where better performance was the most prominent, the imprecise definition of the local maximum was the cause of *DER* errors. Conclusions: Our research clearly indicates that selecting a QRS-detection algorithm based solely on *DER*, *Se*, and *PPV* detection statistics may be incorrect. Two equally important detection quality parameters are the change in the *DER* error rate with tightened requirements of jitter and robustness of the detection statistics *DER*, *Se*, and *PPV* to noise level variations (algorithm’s detection points immunity to noise).

## 1. Introduction

QRS complex detection is a crucial first processing step in almost any automated ECG analysis [1]. The end result of ECG signal processing includes heart rate determination, heart rate variability determination, heartbeat classification, ECG signal fiducial points determination, on-line patient heart monitoring, and other applications. Hence, the accuracy of QRS detection is of primary importance as any errors from the QRS detection stage will be magnified or propagated to consecutive processing steps negatively influencing the end result of the whole processing chain.

The techniques for automatic QRS detection have been actively developed since the 1950s, and many approaches have been proposed [2,3,4], alongside the techniques for heartbeat classification and automated ECG analysis [5,6,7,8,9,10]. The main difficulties faced by QRS detectors are the wide variety of ECG waveforms, especially in cases of heart disease, and various types of interference present in the ECG signal. There are also technical limitations and considerations influencing the effectiveness of QRS detection: sampling rate, ADC resolution, power consumption considerations (especially important for mobile and wearable ECG acquisition systems) [3,4], hardware platform processing capabilities (especially important for real time operation) [3,4].

Despite the long history of research, only recently, the authors have pointed out that, in addition to the standard statistics of correctly detected QRS complexes, an important feature of the detector is the precision of indicating the temporal location of the detection point, and it is most desirable to maintain high accuracy regardless of the noise level. High temporal precision makes fiducial points directly usable for (1) comparing R-waves in heart beats classification process, (2) calculations of rhythm variability parameters such as HRV and HRT, (3) assessments of S-T segments for ischemia monitoring, and many others. No need for additional resynchronization procedure (such as [11]) is also beneficial to reduce the computational complexity, execution time and power requirements.

In [12], we presented an analysis of the effect of detector time tolerance (DTT) on the detection statistics of QRS complex detectors. In [13], we compared four algorithms, including one of our own, in terms of the sensitivity of their detection statistics for individual heart beat morphologies to gradually increase accuracy requirements in the presence of noise with different signal-to-noise ratios (SNRs). Overall, for all four analyzed detectors, the QRS detection accuracy decreased with increasing noise levels and tightening DTT values. It was also shown that the QRS detection accuracy depends on the heartbeat type and is specific to each detector, with some detectors performing better for a given heartbeat type.

In the current work, our objective was to focus on the specific case of the modern implementation of the Pan Tompkins algorithm [14] available on GitHub version 1.3.5 [15] and described in [13] as Alg-4. Following intuition and fragmentary results obtained in [13], we hypothesize that the temporal stability of detection points (i.e., the reproducibility of detection points at the same locations in QRS complexes of the same type) deteriorates linearly with an increasing noise level (i.e., decreasing SNR). To this end, we increased the density of added noise levels and the expected accuracy of true positive detection. In addition to the comprehensive analysis performed for all records in the ECG database within the DTT range where this influence was greatest, we analyzed individual records’ contributions to change in the total DER. Surprisingly, for some records, adding controlled noise to the ECG signal improves the detection statistics of the Alg-4 algorithm for certain DTT values. We therefore analyzed the step-by-step execution of the algorithm for specific strips of the database record with the greatest influence to pinpoint the computational procedure used in Alg-4 that results in the observed non-linear and non-monotonic relation.

Regarding the specific contribution of the present paper, (1) we stress that applying the temporal tolerance analysis may turn over QRS detectors’ rankings based solely on detection statistics, and (2) we suggest revisiting any research related to temporal ECG parameters (such as HRV) based on inaccurate QRS-detection algorithms.

The remainder of this paper is organized as follows: In Section 2 we will first define benchmarks for detection performance. Then, we will look at known schemes for noise removal and known techniques for correcting the location of the R Peak. We analyze how others rate the influence of noise. With Section 3, we propose a method of how to systematically add artificial noise and what its properties should be. We then, in Section 4, analyze whether there is a monotonic relation between an increase in the noise level and the degradation of detection performance. We will see that this is not always given. As this is a surprising result, we dig for the root cause of this strange behavior. Based on our findings, we propose adjusted criteria for characterizing the detection performance of a QRS detector.

## 2. Related Work

QRS detection is the first step in automatic ECG analysis; it is followed by additional blocks necessary to achieve the goal of signal processing. This can be HRV parameter analysis, fiducial point determination, heartbeat classification, etc. All blocks and components should fit with each other, and there are many aspects to achieve the goals of ECG signal processing; therefore, we start this section with an overview of classical and new metrics to assess the QRS detection accuracy (and specifically temporal detection accuracy), followed by the following: noise-removal techniques, corrections of R-peak detection points, HRV accuracy, and finally the influence of noise on detection accuracy.

### 2.1. QRS Detection Benchmarking

The effectiveness of QRS complex detection is typically assessed using a classic binary classification process [2,16]. Reference beat labels (database R peak annotations) are paired with algorithm-produced beat labels (R peaks identified by the algorithm). In order to pair the labels, the maximum time difference between reference the beat label and the algorithm beat label must not exceed a specific time window—the detection time tolerance. In [16], detection time tolerance is specified at 150 ms, whereas in the literature, there are various values: 27.8 ms [17] (ten times sampling period, for MIT-BIH AD [18,19] it is 10 × 2.78 = 27.8 ms) and from 60 ms to 160 ms [12].

Pairing the algorithm’s beat label with the corresponding reference beat label defines binary classification as follows: True Positive (*TP*): a paired reference beat label and algorithm beat label within the detection time tolerance, False Positive detection (*FP*): algorithm beat label without a paired reference beat label, False Negative detection (*FN*): reference beat label without a paired algorithm beat label. The *TP*, *FP*, and *FN* values are then used to calculate the binary classifier metrics: Detection Error Rate (*DER*), Sensitivity (*Se*), Positive Predictive Value (*PPV*), and *F*_1_-score (*F*_1_), as follows:

(1) DER=(FP+FN)/(TP+FN)

(2) Se=TP/(TP+FN)

(3) PPV=TP/(TP+FP)

(4) $$F_{1}=2\cdot PPV\cdot Se/(PPV+Se)$$

where:

*FP*—False Positive,
*FN*—False Negative,
*TP*—True Positive.

Recently, Porr and Macfarlane [17] proposed a new QRS -detection accuracy benchmarking measure, which integrates the detection time tolerance with the *F*_1_-score. They recognized that due to the wide detection time tolerance (for example 150 ms) many QRS-detection algorithms proposed in the literature achieve *F*_1_-scores very close to 100%. With small differences in accuracy results, it is not possible to realistically compare and differentiate the proposed QRS-detection techniques. The authors proposed a new benchmarking measure called *JF*. The process to calculate *JF* consists of several steps. The first step is the determination of *TP_JF_*, *FP_JF_*, and *FN_JF_*. The pairing for calculating *TP_JF_*, *FP_JF_*, and *FN_JF_* is performed without a set width window of detection time tolerance. The basis for pairing is not the window of fixed width (for example 150 ms) but proximity; the nearest algorithm beat label and reference beat label are paired. Any unpaired algorithm beat label between two matched pairs is considered *FP_JF_*, and any unpaired reference beat label between two matched pairs is considered *FN_JF_*. All matched pairs are counted as *TP_JF_*. For all *TP_JF_* over the database record, the average time distance between reference and algorithm labels is calculated and is called the average temporal jitter $\bar{∆}$. As a next step the average temporal jitter is converted into a performance measure between 0 and 100%:

(5) $$f(\bar{∆})=\frac{1}{1+\frac{\bar{∆}}{12\;\mathrm{ms}}}$$

According to (6), the average temporal jitter of 12 ms will produce a performance measure of 0.5 or 50%. As a next step, the *F*_1*JF*_-score is calculated according to (4) with *TP_JF_*, *FP_JF_*, and *FN_JF_*. Finally, the *JF* is calculated:

(6) $$JF=F_{1\;JF}\cdot f\left(\bar{∆}\right)\cdot 100\%$$

In our opinion, the proposed evaluation *JF* has a strong point of integrating temporal accuracy. It can be criticized for joining *Se* and *PPV* into one metric F1 neglecting that FP and FN have vastly different impacts on the downstream processing chain. It can be solved by defining the *JSe* and *JPPV* in an analogous way as *F*_1_ is converted into *JF*.

### 2.2. Noise Removal

Noise removal is often the first step in the QRS-detection algorithm. The authors in [20] describe the types of noise present in the ECG signal then review existing denoising and preprocessing techniques. Then, they review data transformation and detection techniques and list 15 various ECG signal test databases followed by noise parameters, detection accuracy evaluation parameters. The goal of removing the noise from the ECG signal is to improve the accuracy of QRS detection. As there are many types of noise, the study showed that there is no single technique that is effective for all noise conditions. Also, each denoising operation has some impact (distortion) on the underlying ECG signal. And then, review 38 techniques of QRS detection. In conclusion, the authors state that because modern health care requires wearable devices, only a few from reviewed QRS detection algorithms have been tested in a mobile scenario, a noise and power/complexity requirement.

In [21], the authors define and review six main domains of noise-removal techniques: Empirical Mode Decomposition (EMD), Deep-learning autoencoder models (DAEs), wavelet-based models (WT), Sparsity-based models, Bayesian-filter-based models, Hybrid Models. Describe classification of noise into four types: base-line wander, power-line interference, muscle artefacts, channel noise. The types of noise are described and techniques of removal alongside their performance. In summary, the authors give their assessment of which type of filter is best for which type of noise.

In contrast to numerically complex methods proposed in [21,22], the authors present simple practical digital filter implementation to suppress power grid noise using integer coefficients filters. The proposed filter does not introduce the ECG shape deformations and can be used in power-restricted mobile ECG analysis devices.

### 2.3. Corrections of R-Peak Detection Points

The temporal accuracy of R-peak detection is of primary importance for HRV analysis. The authors in [23] observed that filtering in the preprocessing stage introduces time lag in the filtered signal, with delay equal to the group-delay of employed filters. Further, they observed that many algorithms set the time location of the R-peak at the maximum amplitude of the filtered signal, resulting in constant per beat slackness. Many QRS-detection algorithms do not provide compensation for this slackness, so the authors propose compensation as a first step to improve QRS-detection accuracy. As a second step to improve the QRS-detection temporal accuracy, the authors propose a process called the Slackness Reduction Algorithm. After locating the approximate location of the R-peak in the filtered signal, the algorithm searches in the original ECG signal for the more accurate location of the R-peak by searching for the highest absolute differential maximum in the vicinity of the maximum found in the filtered signal. The authors evaluated the results of this modification on three algorithms. They found that the average slackness of this algorithms for MIT-BIH AD was 9 ms for normal beats and 13 ms for abnormal beats. With the Slackness Reduction Algorithm, they reduced the average slackness to 4 ms and 7 ms, respectively.

The sampling interval influences the accuracy of the R-peak location necessary for precise HRV measurements. For example, when the ECG signal is sampled with an interval of 8 ms (*f_s_* = 125 Hz) without modification, that would be a maximum accuracy of HRV. However, it is still possible to maintain the accuracy of the R-peak location on the level of 1–2 ms for precise heart rhythm measurements with the process proposed in [11]. The author reported that by employing simple calculations (eight multiplications, nine additions, and one division on floating point data) for one R-peak, it is possible to achieve the precision level of 1 to 2 ms. The author employed approximation of the R-wave with the quadratic function with the length of approximation of 32 ms. This way, with little additional computation, it is possible to achieve higher temporal accuracy than the sampling interval.

### 2.4. Heart Rate Variability

Accurate detection of QRS complexes is essential for reliable HRV computation. In [24], the authors analyze how the QRS-detection accuracy of various QRS detectors impacts the errors in HRV metrics. They analyze the performance of eight QRS detectors on ECG data from the Glasgow University Database (GUDB) [25]. The GUDB contains ECG signals from 25 subjects recorded under five conditions: sitting, walking, performing a math test, using a handbike, and jogging. In addition, each ECG record contains two simultaneously recorded versions, one recorded via elastic chest strap and one with ECG electrodes. The ECG data was therefore recorded under vastly different levels of noise and artifacts. The 23 HRV metrics were computed based on the outputs from eight QRS detectors and compared with HRV metrics computed based on database annotations. The results show that some detectors perform better than others and none is the best for all tested conditions. Another result is that the relationship between the QRS-detection accuracy and HRV accuracy is metric-dependent and nonlinear. In summary, the authors call for further research to investigate the impact of signal processing and RR interval correction techniques to further improve the robustness of HRV calculations in real-life noisy settings.

In [26], the authors analyzed the performance of a Modular Analysis System (MEANS) to assess its accuracy in heartbeat classification and to study the effects of undetected non-sinus heart beats on HRV parameters. The ECG data used in the study consisted of 20 min ECG records from 1674 patients from the CARLA study (CARdiovascular disease, Living and Ageing in Halle). The heartbeat classification was performed, into three categories: normal sinus rhythm, supraventricular systoles, ventricular systoles. The MEANS system correctly classified 99% of all beats in the study. Compared to reference data, the MEANS system gave a higher SDNN on average (4.9%), and other HRV parameters were also overestimated (from 6.5% to 29%). On the other hand, the results from MEANS did not substantially change the association of HRV and CVD risk factors. The authors also observed that one or few misclassified N-type heartbeats mean large changes in HRV values; at the same time, many false N-type heartbeats lead to small effects on the HRV. In summary, the authors see the need for further improvements in detecting supraventricular systoles or alternatively an indication of the difficulty for algorithmic analysis stretches of the ECG that must have an additional visual reading.

The critical influence of the QRS-detection accuracy on HRV calculations is analyzed in [27]. The analysis was performed on all RR intervals (for simplification all RR intervals were taken instead of the standard approach with only NN intervals) using all MIT-BIH AD records with various added levels of artificial accuracy error. The time domain, frequency domain, and selected nonlinear HRV parameters were calculated (in total 12 parameters) for each added accuracy error. The results show that, in general, time domain parameters are resistant to accuracy error with HRV parameter error values from 1.3% to 0.02% for accuracy error σ = 8 ms and with the exception of pRR50 with an error of 15% for same accuracy error, whereas frequency parameters exhibited errors from 38% to 55% for σ = 8 ms. The selected nonlinear HRV parameters are from 0.83% to 7.5%. In summary, the authors express the opinion that the results of HRV depend strongly on many conditions: sampling frequency, type of QRS detector used, QRS classification procedure, ECG device model, type of heart disease (as low variability of time series and arrhythmic behavior will influence the total HRV error).

### 2.5. Analysis of Noise Influence on Detection Accuracy

In [28], the authors analyze the influence of noise on nine different QRS-detection algorithms, which they selected based on low complexity and high performance. Low complexity criteria were that the algorithm could be run on an 8-bit microprocessor, and with high performance criteria, the authors rejected algorithms with poor accuracy performance for input ECG signals with low noise levels. The algorithms were published from 1971 to 1983. The algorithms were as follows: (1) based on amplitude and first derivative: Moriet-Mahoudeaux, Fraden-Neuman, Gustafson, (2) based on first derivative only: Menard, Holsinger, (3) based on first and second derivative: Balda, Ahlstom-Tompkins, (4) based on digital filters: Engelsee-Zeelenberg, Okada. The ECG signal noise sources identified by the authors were as follows: electromyografic, 60 Hz powerline, baseline drift due to respiration, abrupt baseline drift, and composite noise constructed from the mentioned noise sources. The authors created ECG signals with added simulated noise added to the uncorrupted ECG signal at four levels: 25, 50, 75, and 100% of the maximum amplitude. They adopted 88 ms as temporal time tolerance for the TP/FP/FN determination. The authors also measured and reported the changes in detection delay with an increased level of input noise. In summary, the authors stated that noise deteriorated the accuracy of the algorithms and there was no one best algorithm that was superior for all types of noise considered in their study. For electromyografic noise, the best performance was exhibited by algorithms based on the slope and amplitude. For composite noise, the best performance was recorded for algorithms based on digital filters.

The influence of noise on the accuracy of three well-known algorithms was analyzed in [29]: Pan-Tompkins, WQRS, and Hamilton. Algorithm selection criteria were as follows: real time operation and robust performance with input ECG noise. The authors evaluated *TP*, *FN*, *FP*, *Se*, and *PPV* on records from MIT-BIH AD with different levels of added noise coming from baseline wander (BA), muscle artifact (MA), and electrode motion (EM). Noise data was from the MIT-BIH Noise Stress Test Database. The level of input ECG signal added noise was from −12 dB to 12 dB with a step of 3 dB. The three algorithms showed similar accuracy results expressed in *Se* and *PPV* with an input signal with no added noise. The accuracy deteriorated with added noise and for all 48 MIT-BIH records for SNR = −12dB, the results reported by the authors were respectively for Pan-Tompkins, WQRS, and Hamilton:

- BW noise: Se: 99.42%, 94.58%, 98.13%; PPV: 97.21%, 62.45%, 95.74%
- MA noise: Se: 85.94%, 84.71%, 81.74%; PPV: 59.68%, 36.95%, 61.74%
- EM noise: Se: 68.85%, 65.44%, 84.10%; PPV: 42.54, 33.09%, 44.05%

Is summary, the authors observed that added composite noise at −12 dB significantly degraded all analyzed algorithms’ accuracy expressed in *Se* and *PPV*. In detail, the EM had the highest influence on the detection accuracy, followed by MA and BW. The detection temporal tolerance was not specified by the authors, which makes comparisons to other works difficult.

In [30], the authors proposed an R-peak-detection algorithm based on Hilbert transform and evaluated the algorithm accuracy results *Se*, *PP*, and *DER* achieved with ECG signals contaminated with noise. The ECG signals from MIT-BIH NSTD were prepared with five SNR values from 24 dB to 0 dB with a 6 dB step. The authors compared the results with four detectors known from the literature (Pangerec U. et al., Antink C.H. et al., De Cooman T. et al., Vollmer M.) achieving similar or better results. The detection temporal tolerance adopted by the authors was 150 ms. The results of the proposed QRS-detection method show that up to SNR = 6 dB, the influence of noise on the detection accuracy is minimal with *Se* = 98.13% and *PPV* = 96.91%, whereas for SNR = 0 dB, the accuracy deteriorates significantly to *Se* = 78.98% and *PPV* = 75.25%.

The influence of the noise, detection temporal tolerance (*DTT*), and QRS morphology on QRS-detection accuracy was analyzed in [13]. The authors analyzed four QRS detectors known from the literature (Gutiérrez-Rivas R. et al., Reklewski W. et al., Ravanshad, N. et al., Pan-Tompkins). To measure the changes in the QRS-detection accuracy, a scaled amount of MA noise (four levels) was added to MIT-BIH AD records, and the influences of six QRS morphologies and five values of *DTT* were analyzed. The detection accuracy, expressed as a ratio of True Positive/Total Beats (*TP*/*TB*), generally deteriorated with added levels of noise and decreasing values of detection temporal tolerance. For one detector (Pan-Tompkins), there was an unexpected improvement with added levels of noise for certain values of *DTT*. In terms of the influence of the QRS morphology on detection accuracy, the most difficult for accurate QRS detection was V-type (Ventricular beats) for three algorithms and L-type (Left bundle branch block beat) for one algorithm under study. The average (across all *DTT* and QRS morphologies) detection accuracy results expressed as the True Positive/True Negative ratio (TP/TB) for algorithms under analysis were respectively 83.72% to 82.17%, 90.68% to 89.18%, 77.12% to 71.74%, and 62.03% to 70.32% for levels of noise increasing from SNR = “no noise added” to SNR = 3 dB. As mentioned earlier, for the Pan-Tomkins algorithm, there was an improvement observed in *TP*/*TB* with added noise levels from 62.03% to 70.32%.

## 3. Materials and Methods

**Figure 1:**
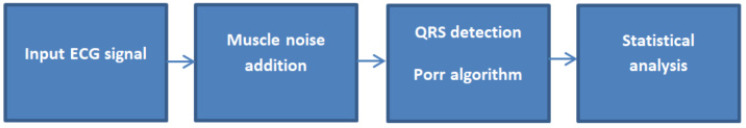
Diagram summarizing the workflow of the tests.

In order to analyze the effects of improving and worsening the *DER* (Equation (1)) with increasing levels of noise and a reduction in the detection tolerance time (*DTT*) on the QRS detector accuracy we have tested algorithm Alg-4 from [13] (Porr algorithm). The tests are carried out for a range of *DTT* values and a range of controlled added muscular noise achieved by mixing muscular noise patterns with MIT-BIH AD records (Figure 1).

First, we reproduced detection accuracy obtained in [13] and expressed it as TP/TB. Next we extended the analysis from [13] with more levels of added noise, expressed as SNR, and conducted the calculations for the same *DTT* range as in [13] but with larger number of *DTT* values in order to reduce result graininess. We switched from measuring the QRS-detection accuracy as the TP/TB ratio to *DER* as it is a more universally employed measure. The *TP*/*TB* and *DER* values here are totals for all 48 MIT-BIH AD records.

To study the influence of an added controlled level of noise to the ECG input signal, we applied a test data set consisting of modified ECG signals from MIT-BIH AD with an added noise signal from MIT-BIH NTSD, a muscle artifact record (MA). We have used all 48 original MIT-BIH AD records, which contain intrinsic noise, and thus, our analysis starts from SNR with an “original” level of noise and not a noise-free ECG record. The added level of SNR has been calculated based on the average power factor of the original record and added noise pattern. The calculation method is described below in the Noise source and mixing section.

The algorithm is tested on the MIT-BIH Arrhythmia Database (MIT-BIH AD) with added muscular noise from the MIT-BIH Noise Stress Test Database (MIT-BIH NSTD) [31]. The tests were conducted on a Dell Latitude E6400, Intel Core2Duo P8400, 2.26 GHz, and 4 GB RAM running with Debian 10.13. Implementation of the algorithms, test tools, and data processing was performed in Python 3.11.2. Plots were created in Jupyter Notebook (server v6.4.12 with Python 3.11.2 [GCC 12.2.0]).

### 3.1. QRS-Detection Algorithm

Algorithm Alg-4 [13] (Porr algorithm) is an implementation of the Pan Tompkins algorithm. It was selected as a study case because of being the most often cited reference algorithm originating from 1985. The original algorithm preprocessing chain consisting of band pass filtering, differentiation, squaring, and the window moving average is retained as the ‘feature function’. The feature function is further analyzed to find local extrema. When a given local extrema surpasses the threshold value, then the local extrema becomes the algorithm R-peak detection and the algorithm enters a 300 ms waiting state, as it is not possible physiologically for the next R-peak to occur within 200 ms; this implementation extends this waiting state to 300 ms. After the pause, the algorithm resumes the local extrema search and comparisons with the threshold value. The searchback mechanism, a adjustment of the threshold value based on regular and irregular heart rates, is implemented.

### 3.2. Detection Time Tolerance Range

The range of acceptable tolerance (*DTT*) tested in this work is presented in Table 1.

### 3.3. Noise Source and Mixing

In order to prepare versions of test ECG records with various levels of noise, we have used the upper channel signal from the MIT-BIH AD and added a noise signal from the MIT-BIH NSTD [31] multiplied by adequate scaling factors. For wearable applications and due to the omnipresence of muscle artifacts, we decided to use a “muscle artifact” (MA) record from the MIT BIH NSTD. According to physionet.org, MIT-BIH NSTD is a database related to MIT-BIH AD and includes 12 half-hour ECG recordings and 3 half-hour recordings of noise typical in ambulatory ECG recordings [31]. The three noise records were assembled from the recordings by selecting intervals that contained predominantly baseline wander (in record ‘bw’), muscle (EMG) artifact (in record ‘ma’), and electrode motion artifact (in record ‘em’) [31].

The records from MIT-BIH AD and MIT-BIH NSTD were captured with the same sampling parameters and have the same length. We have used original records from the MIT-BIH AD, and the intrinsic noise is already present in the data and is out of our control. Consequently, mixing additional noise creates a record with additional noise added on top of already existing noise in the particular record. So this will be called the relative signal-to-noise ratio (*SNR*). The relative *SNR* can be calculated based on the root mean square (RMS) of the original record RMSs (MIT-BIH AD) and added noise pattern RMSn (MIT-BIH NTSD record MA).

(7) $$SNR=20\cdot \log\frac{{RMS}_{s}}{{RMS}_{n}}$$

(8) $${RMS}_{s}=\sqrt{\frac{1}{N}\cdot \sum_{i=1}^{N}{{(xs}_{i})}^{2}}$$

(9) $${RMS}_{n}=\sqrt{\frac{1}{N}\cdot \sum_{i=1}^{N}{{(xn}_{i})}^{2}}$$

where:

*xs_i_*—a record from MIT-BIH AD, signal values
*xn_i_*—MA record from MIT-BIH NSTD, noise values
${RMS}_{s}$—root mean square amplitude of signal
${RMS}_{n}$—root mean square amplitude of noise

The levels of noise mixing (SNR) tested in this work are presented in Table 2.

The following procedure has been employed to achieve target relative *SNR* levels as listed in Table 2. In the first step, the RMSs for one selected record from MIT-BIH AD and RMSn for the MA record from MIT-BIH NSTD are calculated according to Equations (8) and (9). Next, we can rewrite Equation (7) to express the required target RMS as tRMS as in Equation (10). In the next step, the scaling factor *m* is calculated. And the last step includes preparing the record with the required target SNR value according to Equation (12).

(10) $$tSNR=20\cdot \log\frac{{RMS}_{s}}{{m\cdot RMS}_{n}}$$

(11) $$m=\frac{{RMS}_{s}}{{RMS}_{n}}\cdot 10^{\frac{-tSNR}{20}}$$

where:

*m*—scaling factor
tSNR—target SNR

(12) $${ECG}_{tSNR}=ECG+m\cdot MA$$

where:

*ECG*—original ECG record from MIT-BIH AD e.g., record 100
*MA*—MA record from MIT-BIH NTSD

The above presented noise-mixing procedure is repeated for all 48 records from the MIT-BIH AD database and for all 18 target noise levels of values specified in Table 2. This produced a total of 864 half-hour test files used to determine the Detection Error Rate that is the main goal of the experiment.

### 3.4. Individual ECG Record Analysis

Typically, the MIT-BIH AD is used as a collection of typical ECG recordings with specific characteristics representing the true-to-life occurrence of individual heartbeat types. In addition to the global, statistical approach, we also analyzed the impact of individual records on the overall DER value. This allowed us to identify cases particularly sensitive to noise and the tight temporal tolerance of detection points.

## 4. Results

This section presents the results of *DER* calculated for the whole collection of 48 MIT-BIH AD files in the full range of the noise level, initially for the full range of *DTT* and next narrowed to the range where the *DTT* curve is the steepest representing the fastest drop of detector performance (Section 4.1). Next, we limit the range of considered noise levels and seek a performance drop file-by-file to detect the MIT-BIH AD record where the detector performance drop is the fastest (Section 4.2). Surprisingly we also found files in which the presence of noise improves detection statistics, in particular file 123 (Section 4.3). Finally, we focused on selected ECG strips in this file to find the reason for this unexpected result.

### 4.1. General Performance of the Detector Under Test

**Figure 2:**
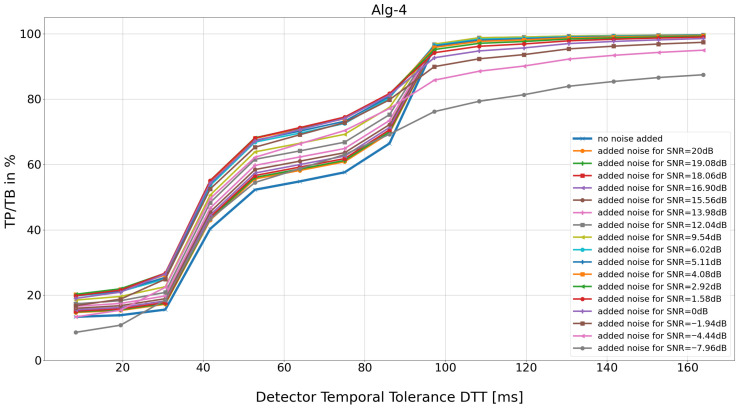
Detection efficiency of the Porr algorithm expressed as TP/TB. The results reflect those obtained and described in [13]. The current calculations were performed for a larger number of *SNR* values (18 values) and *DTT* values (15 values). In [13], 4 *SNR* values and 5 *DTT* values were tested.

**Figure 3:**
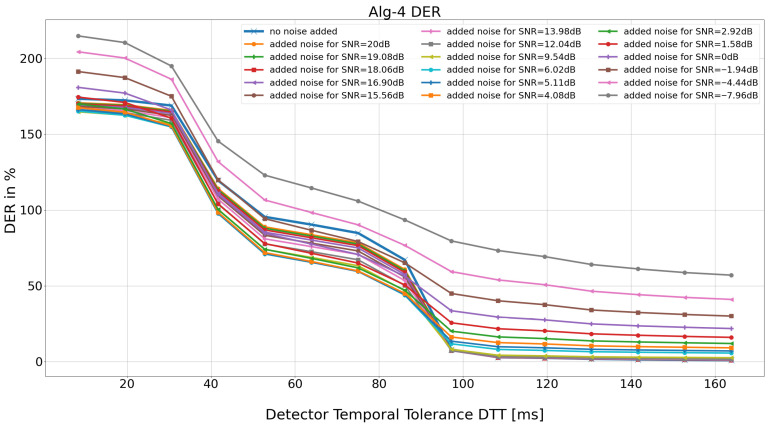
Detection performance results of the Porr algorithm expressed as DER.

For each *DTT* and *SNR* pair, the aggregate detection efficiency (expressed by *TB*, *TP*, *FN*, *FP*, and *DER*) is calculated for 48 records from the MIT BIH AD database (Figure 2 and Figure 3).

**Figure 4:**
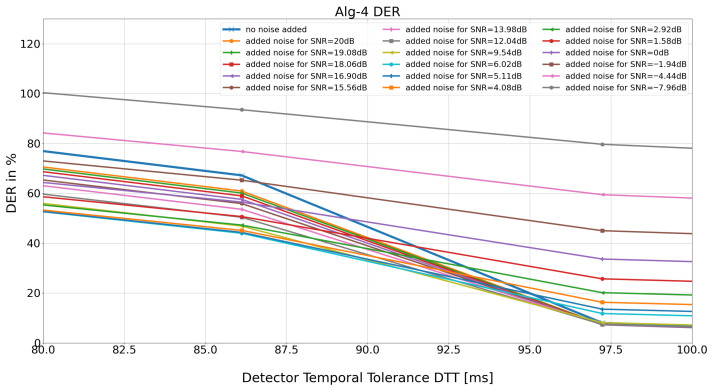
Enlargement of the 35 to 31 sample interval (97.23 ms and 86.12 ms, respectively). For further analysis, the “no added noise” SNR values (blue on the graph) and the noise-added version of the input ECG signal, for added noise up to SNR = 6.02 dB (light blue on the graph), were selected.

Figure 4 displays magnification of Figure 3 within the range of detection temporaltolerance (*DTT*) of 35 to 31 samples (corresponding to 97.23 ms and 86.12 ms), where the detector’s performance statistics drop dramatically.

The *DER* results for individual MIT-BIH AD records for *DTT* = 59, 35, 31, and 3 samples for no noise added and added noise for *SNR* = 6.02 dB are listed in Table A1, Table A2, Table A3 and Table A4 in the Appendix A.

### 4.2. Identification of Most Contributing ECG Files

**Figure 5:**
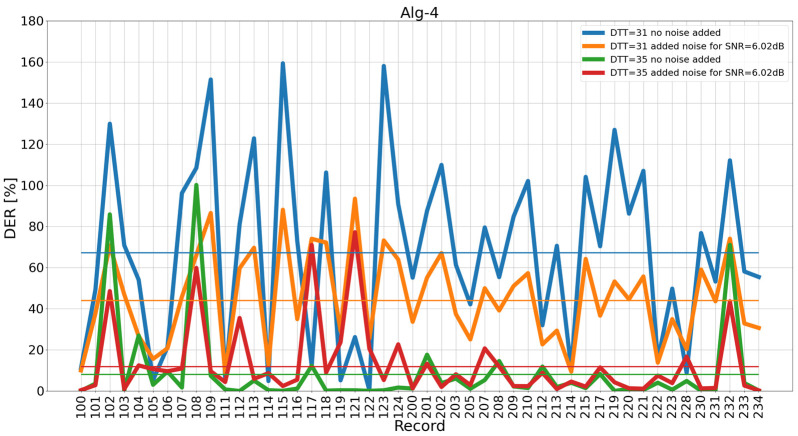
*DER* broken down into individual MIT-BIH AD records with added noise. Total *DER* levels for the 48 MIT-BIH AD records are shown as horizontal lines.

Observing the results in Section 4.1, summarized in Table 3, we asked the following question: which of the 48 MIT-BIH AD records contribute the most to the changes in the total *DER* shown in Figure 3 and Figure 4? Research for an answer led us to study individual record contributions. For this study, we limited the noise level to two values: the “no added noise” (signal number 1 in Table 2) and the noise-added version of input signal (signal number 10 in Table 2), for added noise up to SNR = 6.02 dB. Results of the study are presented in Figure 5.

**Figure 6:**
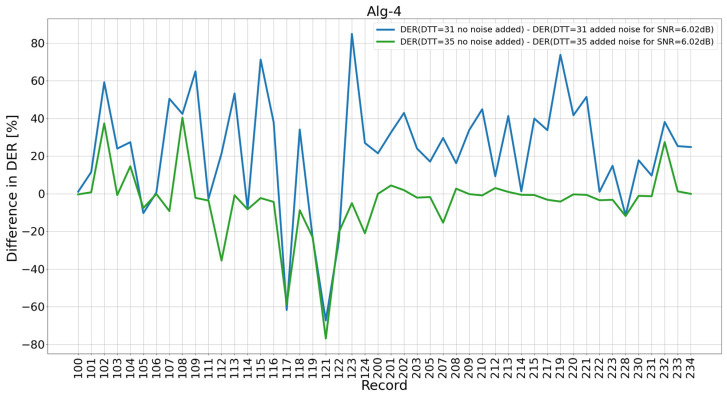
Difference in *DER* for the signal without noise and the signal after adding noise to SNR = 6.02 dB for *DTT* = 31 and *DTT* = 35 of individual MIT-BIH AD records.

**Figure 7:**
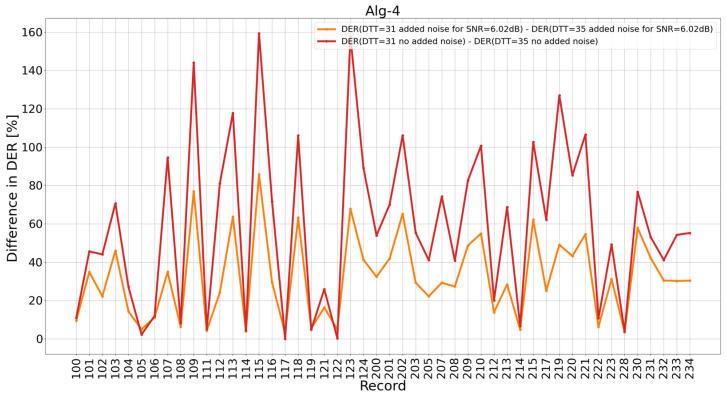
The difference in *DER* of individual MIT BIH AD records. The red color is the DER difference for *DTT* = 31 and *DTT* = 35, both results for the signal without added noise, and the orange color is the analogous difference for the signal with added noise up to the value of SNR = 6.02 dB.

Consequently, we indicated records for which the difference in results without adding noise and after adding noise to SNR = 6.02 dB for fixed *DTT* = 31 and then for fixed *DTT* = 35 was the most important (Figure 6). Moreover, we indicated records for which the change in *DER* after moving from *DTT* = 35 to *DTT* = 31 at a constant *SNR* is the most significant (Figure 7). In Figure 7, it can be seen that the biggest differences in *DER* are for 115, 123, and 109 for both studied *SNR* levels (no added noise and noise up to 6.02 dB).

In Figure 6, it can be seen that the input signal with added noise improves the results the most for record 123 (the difference in DER is approximately +85), followed by record 219 (the difference in DER is approximately +70), and then records 115, 109, and 102. At the same time, it can be observed that for two records, the signal with added noise causes a significant deterioration in the DER result: for 121 (the difference in DER is approximately −70) and 117 (approximately −60). A decrease in detector performance with a growing noise level was somewhat expected; however, the opposite case is worth further studies. To this point, record 123 will be investigated to find a plausible reason for the improvement in the DER after adding noise.

### 4.3. Fiducial Points Instability in File 123

**Figure 8:**
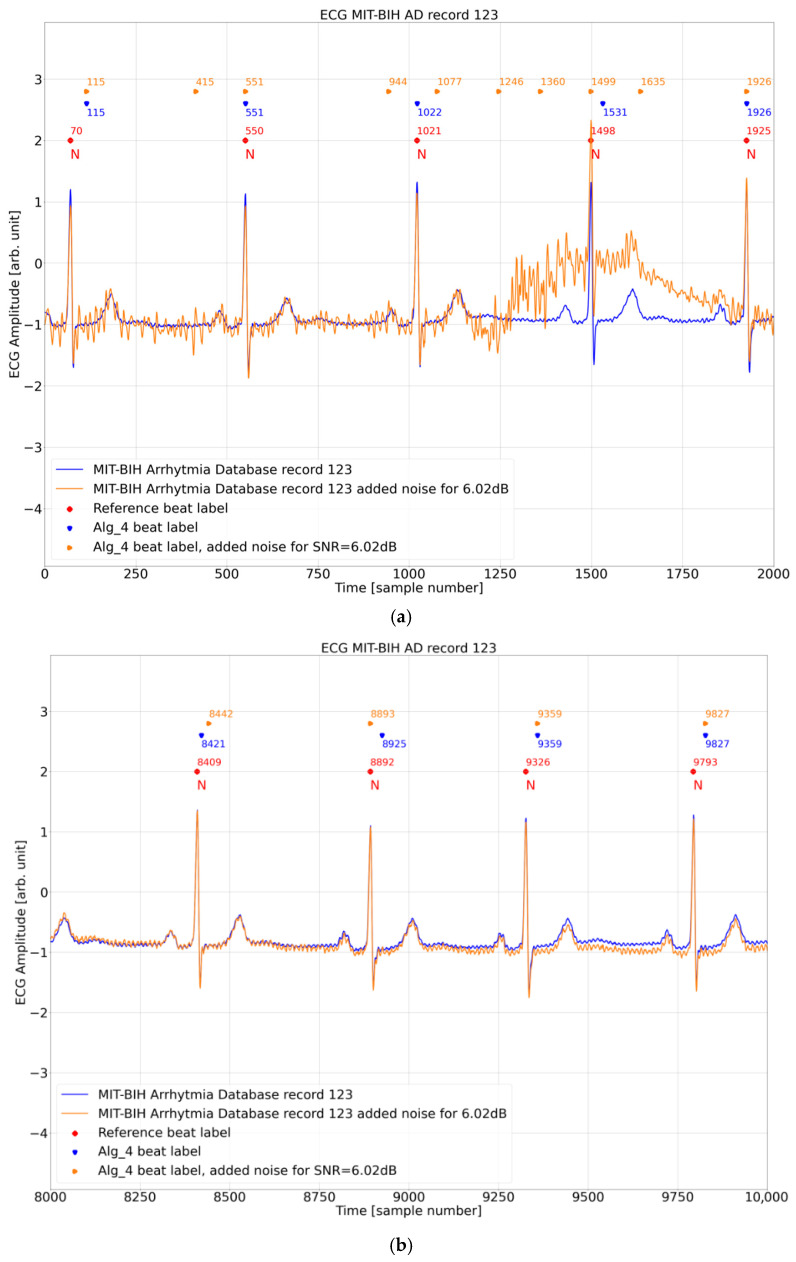

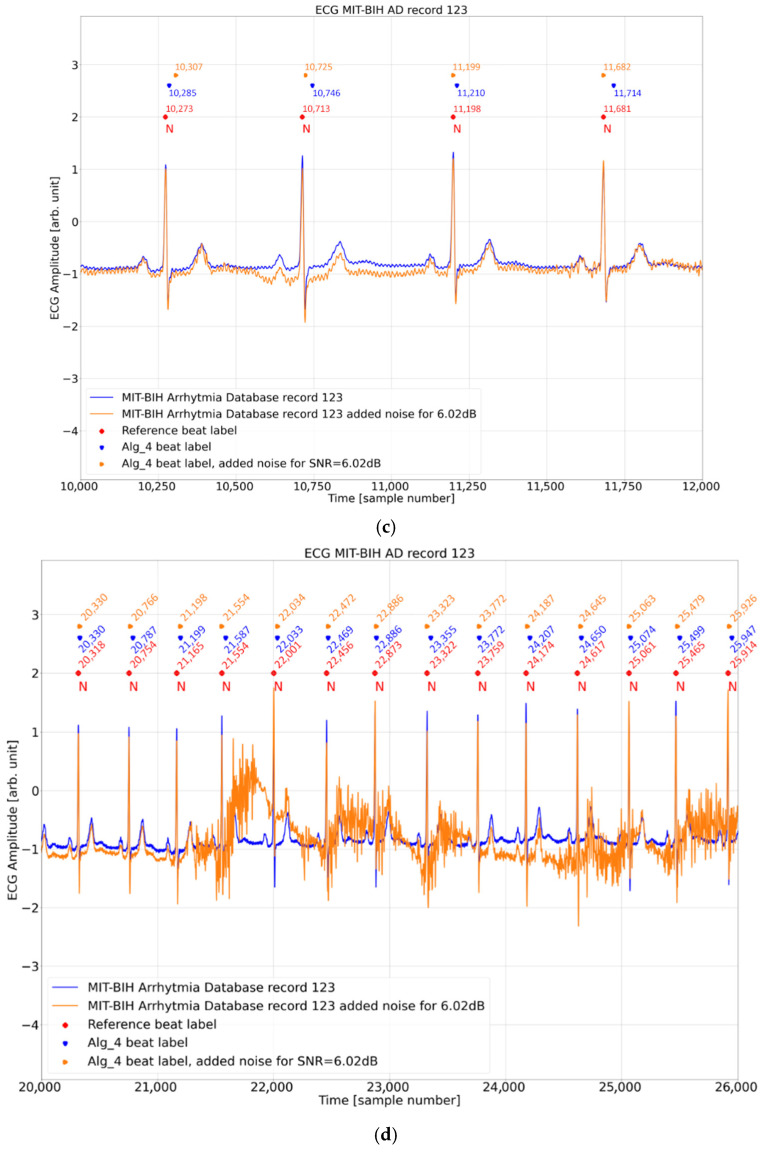
(**a**). Beginning of record 123 with marked detection points of the algorithm for the input signal without added noise and for the added noise for SNR = 6.02 dB and annotations from the MIT BIH AD database. (**b**). Record 123 strip between samples 8000 and 10,000 with marked detection points of the algorithm for the input signal without added noise and for the added noise for SNR = 6.02 dB and annotations from the MIT BIH AD database. (**c**). Record 123 strip between samples 10,000 and 12,000 with marked detection points of the algorithm for the input signal without added noise and for the added noise for SNR = 6.02 dB and annotations from the MIT BIH AD database. (**d**) Record 123 strip between samples 20,000 and 24,000 with marked detection points of the algorithm for the input signal without added noise and for the added noise for SNR = 6.02 dB and annotations from the MIT BIH AD database.

Surprisingly, adding noise may improve the fiducial points stability (i.e., reduce *DER*). In file 123, it can be observed that for the input ECG signal without added noise, some of the algorithm’s detection points are shifted by approximately 30 samples. Annotations from the MIT BIH AD database for time values on the *x*-axis are equal to the following:

- 70 samples (115 − 70 = 45 samples of shift),
- 1498 samples (1531 − 1498 = 33 samples of shift),
- 8892 samples (8925 − 8892 = 33 samples of shift),
- 9326 samples (9359 − 9326 = 33 samples),
- 9793 samples (9827 − 9793 = 34 samples),
- 10,273 samples (10,285 − 10,273 = 12 samples),

Figure 8a–d show detailed fiducial point timing in these particular strips of the 123 record.

### 4.4. Internal Operation Analysis

**Figure 9:**
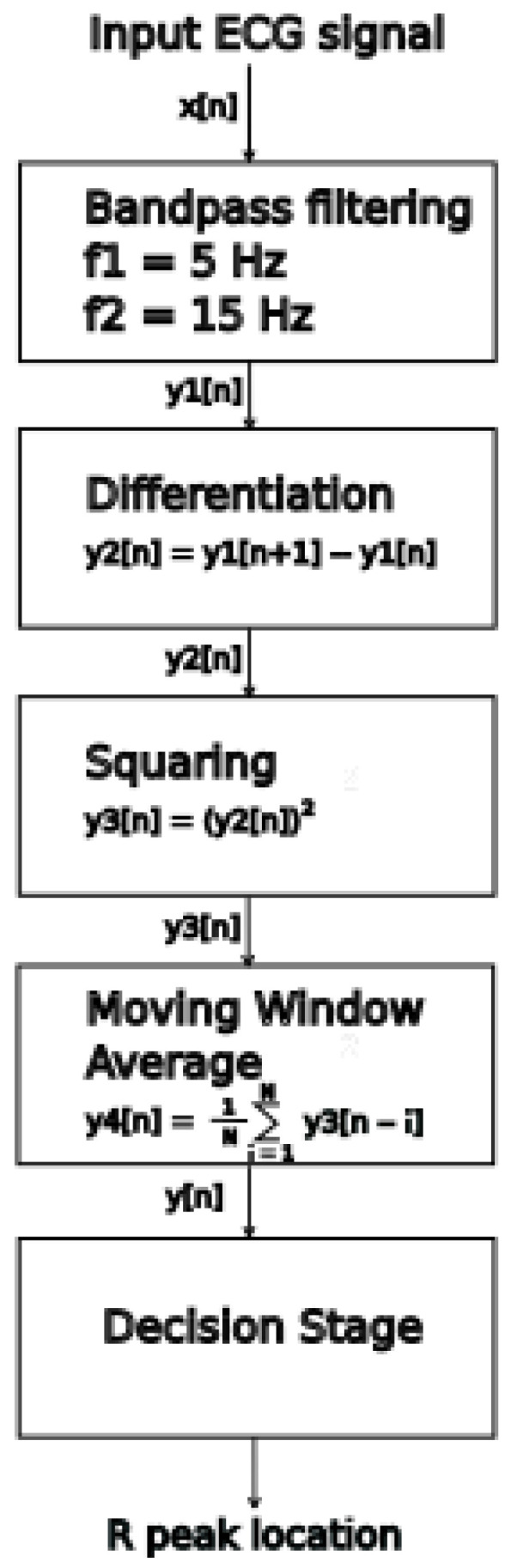
Porr algorithm (Alg-4) flow diagram (based on [13]).

**Figure 10:**
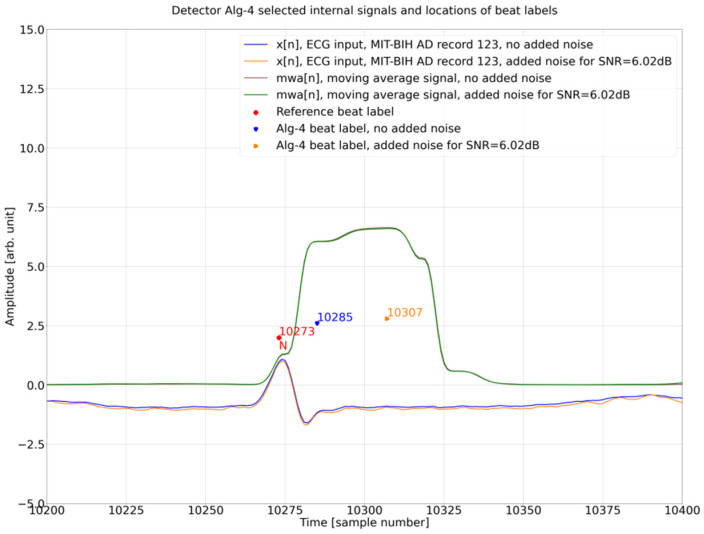
Detector Porr algorithm selected internal signals and locations of beat labels, time period from sample number 10,200 to 10,400.

To shed light on the background of this surprising result (Section 4.3, see particularly Figure 8c with a relatively clean ECG), we analyzed, in greater detail, the Porr algorithm (Figure 9) internal operation for two detections related to database annotation at sample 10,273 and 10,713 for the ECG input signal with no added noise and added noise for SNR = 6.02 dB. For the reference beat label at 10,273, algorithm detection for the ECG input signal with no noise added at 10,285 is close to the annotation with a delay of 12 samples, whereas for the reference beat label at the 10,713 algorithm detection for the ECG input signal with no noise added at 10,725, it is close to the annotation with a delay of 12 samples (Figure 8c). These two beats represent the two opposing results and are thus the subject of further analysis. As can be seen in Figure 10, in this excerpt, there are three cases (detections 2, 3, 4) where the Porr algorithm beat labels for the input ECG signal with added noise are closer to the reference beat label than the beat labels for the input ECG signal with no noise added and one case where detection for no added noise is closer to the reference beat label.

**Figure 11:**
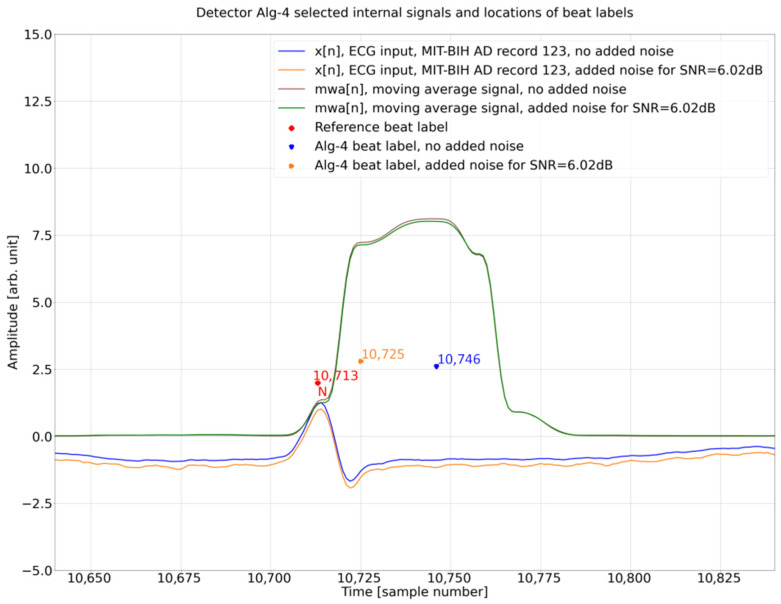
Detector Porr algorithm selected internal signals and locations of beat labels, time from sample 10,640 to 10,840, amplitude of mwa[n] signal magnified to highlight the shape.

Further analysis is presented in Figure 10 and Figure 11, below which an additional signal is presented, the output of the Porr algorithm preprocessing stage—Moving Window Average—mwa[n] signal.

When comparing Figure 10 and Figure 11 it can be noted that in both of these figures, the mwa[n] feature signal (final preprocessing signal before decision stage) looks very similar between the figures and between the two versions of the ECG input signal—the one without added noise (brown line) and the one with added noise (green line). At the same time, the order of algorithm beat labels versus the reference beat label in both figures is different. In Figure 10 the beat label for the input signal without noise is closer to the reference beat label, whereas in Figure 11, the beat label for the input signal with added noise is closer to the reference beat label.

To find out why it is a case, we have analyzed the decision stage in the Porr algorithm. There are two crucial moments:

- finding the peak in the mwa[n] signal,
- checking if the mwa[n] signal value at the found peak is greater than the algorithm detection threshold

The above operations are conducted in the below instructions: Instruction (2) is looking for local maxima in the mwa[n] signal; this is performed for all samples in the ECG record, Instruction (1). Next, for each local maxima found called a ‘peak’, the condition is checked if the signal mwa[n] at the peak is greater than threshold_I1 and if the previously found R peak was more than 108 samples (300 ms) earlier, the refractory condition. If that is true, then, the location found is assigned the algorithm beat label (Algorithm 1).
**Algorithm 1.** Porr algorithm snippet performing peak detection.for i in range (1, lenght_of_ECG_record):                        (1)
   if mwa[i-1] < mwa[i] and mwa[i + 1]<mwa[i]: # if True -> peak is detected   (2)        if mwa [i] > threshold_I1 and (peak-signal_peaks[−1]) > 0.3·fs:      (3)        # second condition checks if previous        # peak is more than 108 samples        # earlier (300 ms refractory blanking).        # if True -> peak is R peak (algorithm        # label) # if False -> peak is a noise peak

Analyzing, in detail, Table 4 in connection with Figure 10, it can be noted that the algorithm-detection delay is smaller for the ECG input signal with no added noise as compared to noise added. This situation can also be observed in the Figure 8b database reference at sample number = 8409, the algorithm beat label for no added noise at 8421, and added noise of 6.02 dB at 8442 and in the Figure 8c database reference at 10,273, algorithm beat label for no added noise at 10,285, and added noise of 6.02 dB at 10,307.

Analyzing, in detail, Table 5 in connection with Figure 11, it can be noted that the algorithm-detection delay is smaller for the ECG input signal with added noise as compared to no noise added. This situation can also be observed in the Figure 8b database reference at sample number 8892 and Figure 8c database reference at sample number 10,713, 11,198, and 11,681.

## 5. Discussion

A regular QRS detector with large jitter of detection points needs additional procedures to center (i.e., synchronize) the QRS waves e.g., parabola fitting. The purpose is twofold: (1) provide a fiducial point to start time-based calculations of parameters (such as HRV) or amplitude measurement points (such as ST slope) and (2) provide a reference for a cross-correlation-based comparison of signals (for clustering and classification). Two approaches have been presented in the literature: either such centering was performed and precise R-points were calculated at the cost of additional computational load, or the centering was omitted saving machine cycles at the cost of parameters of only approximate quality. Our paper identifies another alternative: having a low complexity low jitter QRS detector robust to noise. In particular, the presence of noise together with low power requirements is typical for wearables; our findings may thus be of particular interest in pursuit for high quality ECG parameters from wearables.

The results obtained show that for almost all *SNR* values, when the *DTT* value is reduced from *DTT* = 97.23 ms to *DTT* = 86.12 ms (i.e., from *DTT* = 35 to *DTT* = 31 samples), there is a sharp drop in performance (i.e., rise of total *DER*) for all 48 MIT-BIH AD records. This is an interesting novelty in the methodology, because it would allow the detector to be examined as a black box, i.e., without knowledge of its structure. Regarding the detector under test, a delay (inaccuracy of the ‘R’ reading) of 80–100 ms is quite significant. The largest drop in the value of DER was observed for two SNR values: SNR = ‘no added noise’ and SNR = 6.02 dB (ratio of average amplitudes $\frac{A_{signal}}{A_{noise}}=2$, i.e., the average amplitude of the useful signal is 2 times greater than the added noise). The amount of delay of this particular algorithm suggests that fiducial points, being its results, should rather be corrected before use in time-related ECG procedures. This statements challenges many papers in which the authors use the QRS-detection series directly for calculations of HRV, heart beat classifications, or estimations of the S-T segment.

In [32], we proposed a multiplierless QRS detector working fast (i.e., with minimum computational burden), precisely (i.e., with excellent detection statistics), and with far less delay (i.e., maintaining good results with less tolerant assumptions). We compared it to several other low-delay QRS detection methods developed recently.

In this paper, we examined the most popular implementation of the most popular QRS-detection algorithm, developed by Pan Tompkins [14], implemented by [15] and available on GitHub. Based on citation statistics, this implementation is considered the most frequently used as a ‘black box’ by the developers of ECG algorithms with a wide range of complexity. To shed more light on the implementation quality, we examined it, applying more true-to-life signal conditions and possibly more thoroughly than anyone previously, using the most accessible database of MIT-BIH AD records [19,20].

- First, we generated regular statistics of detection performance (incl. Acc, PPV, DER etc.).
- Next, we examined how detection-performance statistics decrease with increasing timing accuracy requirements by narrowing the range of temporal tolerance (*DTT*), i.e., allowed time between detection and reference points. We concluded that this algorithm’s results are not directly applicable for time series analyses of ECG, such as HRV, QT, S-T, etc.
- Further, we examined how increased noise levels affect the stability of the detection point. We found that, as expected, in most cases, higher noise levels lead to greater inaccuracies. However, we also observed situations (files: 123, 219, 115, 109, and 102) where higher noise levels resulted in improved detection statistics.
- We found several outlying cases and further seek the file in which this surprising situation was most pronounced. For selected ECG segments in file 123, we analyzed the algorithm’s execution step by step. We concluded that the source of the unexpected behavior is the ambiguous implementation of the local maximum definition applied to the feature function.

**Figure 12:**
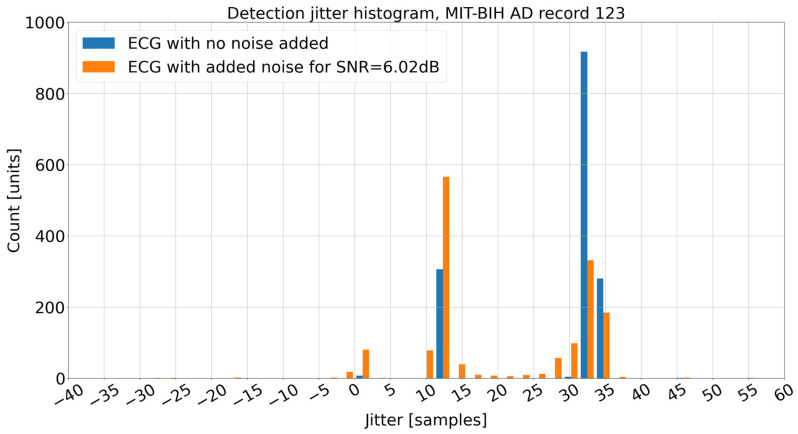
Detection jitter histogram.

The observed phenomena of *DER* improvement based on the ECG input signal with added noise for SNR = 6.02 dB are caused by the local maximum detection mechanism used in the Decision Stage (Figure 9). The local maximum that is a candidate for the R break is searched in the mwa[n] signal. In idealized relationship [33] between the QRS complex and mwa[n] signal, the R peak, occurs approximately in the middle of the rising slope of mwa[n] (Figure 10 and Figure 11) (the time shift noticeable in Figure 10 and Figure 11 is due to group delay of the bandpass filter and a delay in differentiation and moving window average operations, Figure 9). In theory, the Porr algorithm is supposed to detect the first peak as soon as the rising slope ends (Figure 10 and Figure 11). In practice, due to the requirement for the local peak, the preceding and following samples have to have lower values, and considering that the mwa[n] signal is the time-averaged signal over 54 samples (150 ms), this condition for the local peak is often not met around the beginning of the plateau, and the detection of the peak often occurs later during or at the end of the plateau of the mwa[n] signal. This delay has been observed in this work to be about 33 samples (approx. 92 ms), which is a significant number and can be observed in Figure 12. Adding noise for *SNR* = 6.02 dB reduces the detection jitter by moving a significant number of detections to about 13 samples (approx. 36 ms); this can be observed in the histogram in Figure 12.

Adding noise increases variability of the mwa[n] signal, which often results in earlier detection of the local extrema, reducing the jitter, as has been observed in Figure 11 and Table 5, which in turn translates to improved *DER* results for a given *DTT*. Of course, for the tested algorithm, the *DER* is already poor at *DTT* = 31 samples (Table A3), but the fact still remains that the *DER* for the added noise of 6.02 dB is lower than the *DER* for no added noise for *DTT* = 31 and lower values of *DTT*.

Our research points out that Pan Tompkins as the QRS-detector reference has a limitation not described in the existing literature. Current detectors are compared with a flawed reference; then, the comparison is meaningless, and science is not advanced. Despite better results, we do not find a better commonly used reference in the literature. Moreover, in different variants, the Pan Tompkins algorithm is widely used as a front-end black-box procedure by researchers investigating further steps of the ECG-processing chain.

Results of this paper are potentially interesting as an example of detailed testing of the QRS-detector procedure, where most authors only provide selected detection statistics (*DER*, *PPV*, *Se* without *DTT*, and statistics noise resistance). Both [13] and [17] are consistent in showing that most detectors are converging to 100% accuracy if *DTT* is large enough (i.e., inaccurate pairing is allowed). Our results may also motivate one to revisit other papers focused on further steps of ECG processing, with a carelessly implemented flawed version of the reference QRS detector (Pan Tompkins) as a black-box ready-to-use procedure.

It is worth a remark that an imprecise definition of the local maximum was already noticed in an early improvement of the Pan Tompkins [14] algorithm, known as the Hamilton Tompkins method [33]. In the algorithm implementation selected in our study, the local peak was searched in the moving average signal (mwa[n]) with a series of if–then conditions and depending on the expected signal properties (sample to sample value changes), which are not met, thus omitting the maximum at its true position. The conclusion from our research is much more general and does not apply only to the Alg. 4 detector. Thorough testing of the detector (and virtually any kind of medical interpretive software) in any imaginable conditions helps in revealing software faults of which the effects are rarely revealed but medically important.

## 6. Conclusions

Our research clearly indicates that selecting a QRS-detection algorithm based solely on detection statistics may lead to incorrect results. A significant parameter is the change in statistics as the requirements for the temporal accuracy of the detection point change. Algorithms that maintain high true positive detection statistics, even within narrow time tolerances, enable direct application of their results to time-based ECG analyses such as HRV. Algorithms for which statistics deteriorate rapidly require the use of centering procedures to stabilize the QRS-detection point locations. Another important parameter is the robustness of the detection statistics and the temporal stability of the detection point to noise level variations. Long-term electrocardiogram recordings in normal human life are typically accompanied by variable muscle activity and the influence of external electromagnetic fields, so the QRS detector’s immunity to interference fluctuations is highly desirable.

## Figures and Tables

**Table 1:** Range of *DTT* values tested.

Approach No.	Detector Temporal Tolerance—DTT	
	[Samples]	[ms]
1	3	8.33
2	7	19.44
3	11	30.56
4	15	41.67
5	19	52.78
6	23	63.89
7	27	75.00
8	31	86.11
9	35	97.22
10	39	108.33
11	43	119.45
12	47	130.56
13	51	141.67
14	55	152.78
15	59	163.90

**Table 2:** Range of added noise levels tested.

ECG Input Signal Version No.	$\frac{{\mathrm{RMS}}_{s}}{{\mathrm{RMS}}_{n}}$	$20\cdot \log_{10}\frac{{\mathrm{RMS}}_{s}}{{\mathrm{RMS}}_{n}}$
	[-]	[db]
1	No noise added	No noise added
2	10	20
3	9	19.08
4	8	18.06
5	7	16.90
6	6	15.56
7	5	13.98
8	4	12.04
9	3	9.54
10	2	6.02
11	1.8	5.11
12	1.6	4.08
13	1.4	2.92
14	1.2	1.58
15	1	0
16	0.8	−1.94
17	0.6	−4.44
18	0.4	−7.96

**Table 3:** Detection performance results of the Porr algorithm expressed as *DER* for no noise added and added noise for *SNR* = 6.02 dB. Range of *DTT* from 3 sample (8.33 ms) to 59 sample (163.90 ms) totals for all 48 MIT-BIH AD records. Total DER pooled over all records (TP, FN, and FP added first then total DER calculated).

DTT [Samples]	DTT [ms]	Added Noise [dB]	All MIT-BIH AD Records	TB	TP	FN	FP	Se [%]	PPV [%]	DER [%]
3	8.33	no noise added	TOTAL	109494	14630	94864	94957	13.36	13.35	173.36
3	8.33	6.02	TOTAL	109494	21633	87861	93060	19.76	18.86	165.23
7	19.45	no noise added	TOTAL	109494	15182	94312	94405	13.87	13.85	172.35
7	19.45	6.02	TOTAL	109494	23118	86376	91575	21.11	20.16	162.52
11	30.56	no noise added	TOTAL	109494	15182	94312	94405	13.87	13.85	172.35
11	30.56	6.02	TOTAL	109494	27355	82139	87338	24.98	23.85	154.78
15	41.67	no noise added	TOTAL	109494	44112	65382	65475	40.29	40.25	119.51
15	41.67	6.02	TOTAL	109494	58599	50895	56094	53.52	51.09	97.71
19	52.78	no noise added	TOTAL	109494	57224	52270	52363	52.26	52.22	95.56
19	52.78	6.02	TOTAL	109494	73178	36316	41515	66.83	63.80	71.08
23	63.89	no noise added	TOTAL	109494	60023	49471	49564	54.82	54.77	90.45
23	63.89	6.02	TOTAL	109494	76186	33308	38507	69.58	66.43	65.59
27	75.01	no noise added	TOTAL	109494	63067	46427	46520	57.60	57.55	84.89
27	75.01	6.02	TOTAL	109494	79415	30079	35278	72.53	69.24	59.69
31	86.12	no noise added	TOTAL	109494	72740	36754	36847	66.43	66.38	67.22
31	86.12	6.02	TOTAL	109494	88007	21487	26686	80.38	76.73	44.00
35	97.23	no noise added	TOTAL	109494	105118	4376	4469	96.00	95.92	8.08
35	97.23	6.02	TOTAL	109494	105625	3869	9068	96.47	92.09	11.82
39	108.34	no noise added	TOTAL	109494	107949	1545	1638	98.59	98.51	2.91
39	108.34	6.02	TOTAL	109494	107651	1843	7042	98.32	93.86	8.11
43	119.45	no noise added	TOTAL	109494	108159	1335	1428	98.78	98.70	2.52
43	119.45	6.02	TOTAL	109494	108015	1479	6678	98.65	94.18	7.45
47	130.57	no noise added	TOTAL	109494	108525	969	1062	99.12	99.03	1.85
47	130.57	6.02	TOTAL	109494	108481	1013	6212	99.07	94.58	6.60
51	141.68	no noise added	TOTAL	109494	108751	743	836	99.32	99.24	1.44
51	141.68	6.02	TOTAL	109494	108672	822	6021	99.25	94.75	6.25
55	152.79	no noise added	TOTAL	109494	108894	600	693	99.45	99.37	1.18
55	152.79	6.02	TOTAL	109494	108820	674	5873	99.38	94.88	5.98
59	163.90	no noise added	TOTAL	109494	109002	492	585	99.55	99.47	0.98
59	163.90	6.02	TOTAL	109494	108974	520	5719	99.53	95.01	5.70

**Table 4:** Detector Porr algorithm locations of True-True evaluation of Instruction (2) meaning a local peak in the mwa[n] signal, and True-True evaluation of Instruction (3) meaning a feature signal greater than the threshold and more than 300 ms from the last algorithm detection (beat label), with fill colors corresponding to Figure 10, MIT-BIH AD record 123.

Input ECG Signal No Added Noise						Input ECG Signal with Added Noise for SNR = 6.02 dB				
**sample** **number i**	**mwa[i-1]** **< mwa[i]** **rising slope**	**mwa[i+1]** **< mwa[i]** **falling slope**	**mwa[i]** **> threshold_** **I1[i]** **feature signal** **> threshold**	**(peak-signal_** **peaks[-1])** **> 0.3·fs** **108 samples from last peak (300 ms)**		**sample** **number i**	**mwa[i-1]** **< mwa[i]** **rising slope**	**mwa[i+1]** **< mwa[i]** **falling** **slope**	**mwa[i]** **> threshold_** **I1[i]** **feature signal** **> threshold**	**(peak-signal_** **peaks[-1])** **> 0.3·fs** **108 samples from last peak (300 ms)**
10200	False	False	False	True		10200	False	True	False	True
…						…				
10201	True	True	False	True		10209	True	True	False	True
10222	True	True	False	True		10222	True	True	False	True
…						10226	True	True	False	True
10238	True	True	False	True		10238	True	True	False	True
10246	True	True	False	True		10246	True	True	False	True
10273	True	False	False	True		10273	True	False	False	True
10285	True	True	True	True		…				
10307	True	True	True	False		10307	True	True	True	True
10354	True	True	False	False		10353	True	True	False	False
10364	True	True	False	False		10364	True	True	False	False
10371	True	True	False	False		10370	True	True	False	False
10380	True	True	False	False		10385	True	True	False	False
10382	True	True	False	True		…				
10388	True	True	False	True		10390	True	True	False	False
10400	True	False	False	True		10400	True	False	False	False

**Table 5:** Detector Porr algorithm locations of True-True evaluation of Instruction (2) meaning a local peak in the mwa[n] signal, and True-True evaluation of Instruction (3) meaning a feature signal greater than the threshold and more than 300 ms from the last algorithm detection (beat label), with fill colors corresponding to Figure 11, MIT-BIH AD record 123.

Input ECG Signal No Added Noise						Input ECG Signal with Added Noise for SNR = 6.02 dB				
**sample** **number i**	**mwa[i-1]** **< mwa[i]** **rising slope**	**mwa[i+1]** **< mwa[i]** **falling** **slope**	**mwa[i]** **> threshold_** **I1[i]** **feature signal** **> threshold**	**(peak-signal_** **peaks[-1])** **> 0.3·fs** **108 samples from last peak (300 ms)**		**sample** **number i**	**mwa[i-1]** **< mwa[i]** **rising slope**	**mwa[i+1]** **< mwa[i]** **falling** **slope**	**mwa[i]** **> threshold_ I1[i]** **feature signal** **> threshold**	**(peak-signal_** **peaks[-1])** **> 0.3·fs** **108 samples from last peak (300 ms)**
10640	False	True	False	True		10640	False	False	True	True
10660	True	True	False	True		10642	True	True	False	True
…						10654	True	True	False	True
…						10660	True	True	False	True
10670	True	True	False	True		10670	True	True	False	True
10681	True	True	False	True		10681	True	True	False	True
…						10685	True	True	False	True
…						10697	True	True	False	True
10713	True	False	False	True		10713	True	False	False	True
10714	True	True	False	True		…				
…						10725	True	True	True	True
10746	True	True	True	True		10744	True	True	True	False
10758	True	True	True	False		10758	True	True	True	False
10769	True	True	False	False		10769	True	True	False	False
10793	True	True	False	False		10789	True	True	False	False
…						10797	True	True	False	False
…						10807	True	True	False	False
10815	True	True	False	False		10816	True	True	False	False
10822	True	True	False	False		10823	True	True	False	False
10825	True	True	False	False		…				
10829	True	True	False	False		10828	True	True	False	False
10840	True	False	False	False		10840	True	False	False	False

## Data Availability

The original contributions presented in this study are included in the article. Further inquiries can be directed to the corresponding author.

## Appendix A

**Table A1:** *DER* Results for individual MIT-BIH AD records, and Total, for *DTT* = 59 samples (163.90 ms), no added noise, and added noise for *SNR* = 6.02 dB.

		DTT = 59 Samples (163.90 ms) No Added Noise							DTT = 59 Samples (163.90 ms) Added Noise for SNR = 6.02 dB					
MIT-BIH AD Record	TB	TP	FN	FP	Se [%]	PPV [%]	DER [%]		TP	FN	FP	Se [%]	PPV [%]	DER [%]
100	2273	2272	1	0	99.96	100	0.04		2271	2	3	99.91	99.87	0.22
101	1865	1863	2	5	99.89	99.73	0.38		1863	2	9	99.89	99.52	0.59
102	2187	2187	0	0	100	100	0		2182	5	69	99.77	96.93	3.38
103	2084	2080	4	1	99.81	99.95	0.24		2079	5	1	99.76	99.95	0.29
104	2229	2225	4	29	99.82	98.71	1.48		2223	6	73	99.73	96.82	3.54
105	2572	2564	8	38	99.69	98.54	1.79		2551	21	114	99.18	95.72	5.25
106	2027	2026	1	1	99.95	99.95	0.1		2022	5	32	99.75	98.44	1.83
107	2137	2133	4	0	99.81	100	0.19		2129	8	127	99.63	94.37	6.32
108	1763	1539	224	408	87.29	79.04	35.85		1698	65	508	96.31	76.97	32.5
109	2532	2529	3	0	99.88	100	0.12		2528	4	9	99.84	99.65	0.51
111	2124	2123	1	1	99.95	99.95	0.09		2117	7	50	99.67	97.69	2.68
112	2539	2539	0	0	100	100	0		2528	11	606	99.57	80.66	24.3
113	1795	1794	1	2	99.94	99.89	0.17		1794	1	1	99.94	99.94	0.11
114	1879	1878	1	6	99.95	99.68	0.37		1874	5	98	99.73	95.03	5.48
115	1953	1953	0	0	100	100	0		1953	0	3	100	99.85	0.15
116	2412	2388	24	2	99	99.92	1.08		2385	27	60	98.88	97.55	3.61
117	1535	1534	1	1	99.93	99.93	0.13		1516	19	905	98.76	62.62	60.2
118	2278	2278	0	0	100	100	0		2276	2	92	99.91	96.11	4.13
119	1987	1987	0	1	100	99.95	0.05		1977	10	324	99.5	85.92	16.81
121	1863	1863	0	0	100	100	0		1843	20	1086	98.93	62.92	59.37
122	2476	2476	0	0	100	100	0		2446	30	303	98.79	88.98	13.45
123	1518	1515	3	0	99.8	100	0.2		1511	7	56	99.54	96.43	4.15
124	1619	1619	0	1	100	99.94	0.06		1607	12	265	99.26	85.84	17.11
200	2601	2598	3	4	99.88	99.85	0.27		2598	3	10	99.88	99.62	0.5
201	1963	1913	50	0	97.45	100	2.55		1912	51	7	97.4	99.64	2.95
202	2136	2130	6	2	99.72	99.91	0.37		2131	5	5	99.77	99.77	0.47
203	2980	2919	61	23	97.95	99.22	2.82		2925	55	95	98.15	96.85	5.03
205	2656	2650	6	1	99.77	99.96	0.26		2648	8	12	99.7	99.55	0.75
207	1860	1857	3	8	99.84	99.57	0.59		1846	14	221	99.25	89.31	12.63
208	2955	2932	23	1	99.22	99.97	0.81		2930	25	10	99.15	99.66	1.18
209	3005	3004	1	1	99.97	99.97	0.07		3005	0	2	100	99.93	0.07
210	2650	2624	26	4	99.02	99.85	1.13		2621	29	9	98.91	99.66	1.43
212	2748	2748	0	0	100	100	0		2748	0	1	100	99.96	0.04
213	3251	3250	1	0	99.97	100	0.03		3250	1	1	99.97	99.97	0.06
214	2262	2258	4	0	99.82	100	0.18		2258	4	11	99.82	99.52	0.66
215	3363	3362	1	0	99.97	100	0.03		3362	1	0	99.97	100	0.03
217	2208	2205	3	3	99.86	99.86	0.27		2198	10	87	99.55	96.19	4.39
219	2154	2154	0	1	100	99.95	0.05		2151	3	64	99.86	97.11	3.11
220	2048	2047	1	1	99.95	99.95	0.1		2046	2	5	99.9	99.76	0.34
221	2427	2420	7	1	99.71	99.96	0.33		2421	6	11	99.75	99.55	0.7
222	2483	2482	1	5	99.96	99.8	0.24		2475	8	82	99.68	96.79	3.62
223	2605	2602	3	1	99.88	99.96	0.15		2601	4	34	99.85	98.71	1.46
228	2053	2049	4	24	99.81	98.84	1.36		2039	14	187	99.32	91.6	9.79
230	2256	2256	0	0	100	100	0		2256	0	0	100	100	0
231	1571	1571	0	0	100	100	0		1571	0	1	100	99.94	0.06
232	1780	1779	1	9	99.94	99.5	0.56		1779	1	67	99.94	96.37	3.82
233	3079	3074	5	0	99.84	100	0.16		3077	2	3	99.94	99.9	0.16
234	2753	2753	0	0	100	100	0		2753	0	0	100	100	0
TOTAL	109494	109002	492	585	99.55	99.47	0.98		108974	520	5719	99.53	95.01	5.70

**Table A2:** *DER* Results for individual MIT-BIH AD records, and Total, for *DTT* = 35 samples (97.23 ms), no added noise, and added noise for *SNR* = 6.02 dB.

		DTT = 35 Samples (97.23 ms) No Added Noise							DTT = 35 Samples (97.23 ms) Added Noise for SNR = 6.02 dB					
MIT-BIH AD Record	TB	TP	FN	FP	Se[%]	PPV[%]	DER[%]		TP	FN	FP	Se[%]	PPV[%]	DER[%]
100	2273	2272	1	0	99.96	100	0.04		2269	4	5	99.82	99.78	0.4
101	1865	1833	32	35	98.28	98.13	3.59		1842	23	30	98.77	98.4	2.84
102	2187	1248	939	939	57.06	57.06	85.87		1688	499	563	77.18	74.99	48.56
103	2084	2080	4	1	99.81	99.95	0.24		2072	12	8	99.42	99.62	0.96
104	2229	1941	288	313	87.08	86.11	26.96		2124	105	172	95.29	92.51	12.43
105	2572	2548	24	54	99.07	97.92	3.03		2482	90	183	96.5	93.13	10.61
106	2027	1931	96	96	95.26	95.26	9.47		1943	84	111	95.86	94.6	9.62
107	2137	2117	20	16	99.06	99.25	1.68		2080	57	176	97.33	92.2	10.9
108	1763	972	791	975	55.13	49.92	100.17		1457	306	749	82.64	66.05	59.84
109	2532	2437	95	92	96.25	96.36	7.39		2413	119	124	95.3	95.11	9.6
111	2124	2116	8	8	99.62	99.62	0.75		2099	25	68	98.82	96.86	4.38
112	2539	2539	0	0	100	100	0		2386	153	748	93.97	76.13	35.49
113	1795	1750	45	46	97.49	97.44	5.07		1742	53	53	97.05	97.05	5.91
114	1879	1878	1	6	99.95	99.68	0.37		1844	35	128	98.14	93.51	8.67
115	1953	1952	1	1	99.95	99.95	0.1		1931	22	25	98.87	98.72	2.41
116	2412	2387	25	3	98.96	99.87	1.16		2362	50	83	97.93	96.61	5.51
117	1535	1441	94	94	93.88	93.88	12.25		1433	102	988	93.36	59.19	71.01
118	2278	2276	2	2	99.91	99.91	0.18		2221	57	147	97.5	93.79	8.96
119	1987	1984	3	4	99.85	99.8	0.35		1909	78	392	96.07	82.96	23.65
121	1863	1860	3	3	99.84	99.84	0.32		1677	186	1252	90.02	57.26	77.19
122	2476	2475	1	1	99.96	99.96	0.08		2358	118	391	95.23	85.78	20.56
123	1518	1514	4	1	99.74	99.93	0.33		1502	16	65	98.95	95.85	5.34
124	1619	1606	13	14	99.2	99.14	1.67		1562	57	310	96.48	83.44	22.67
200	2601	2586	15	16	99.42	99.39	1.19		2588	13	20	99.5	99.23	1.27
201	1963	1765	198	148	89.91	92.26	17.63		1811	152	108	92.26	94.37	13.25
202	2136	2093	43	39	97.99	98.17	3.84		2115	21	21	99.02	99.02	1.97
203	2980	2871	109	71	96.34	97.59	6.04		2879	101	141	96.61	95.33	8.12
205	2656	2639	17	12	99.36	99.55	1.09		2620	36	40	98.64	98.5	2.86
207	1860	1813	47	52	97.47	97.21	5.32		1771	89	296	95.22	85.68	20.7
208	2955	2729	226	204	92.35	93.04	14.55		2772	183	168	93.81	94.29	11.88
209	3005	2972	33	33	98.9	98.9	2.2		2970	35	37	98.84	98.77	2.4
210	2650	2620	30	8	98.87	99.7	1.43		2609	41	21	98.45	99.2	2.34
212	2748	2585	163	163	94.07	94.07	11.86		2627	121	122	95.6	95.56	8.84
213	3251	3219	32	31	99.02	99.05	1.94		3235	16	16	99.51	99.51	0.98
214	2262	2215	47	43	97.92	98.1	3.98		2214	48	55	97.88	97.58	4.55
215	3363	3339	24	23	99.29	99.32	1.4		3327	36	35	98.93	98.96	2.11
217	2208	2117	91	91	95.88	95.88	8.24		2120	88	165	96.01	92.78	11.46
219	2154	2154	0	1	100	99.95	0.05		2139	15	76	99.3	96.57	4.22
220	2048	2038	10	10	99.51	99.51	0.98		2036	12	15	99.41	99.27	1.32
221	2427	2418	9	3	99.63	99.88	0.49		2416	11	16	99.55	99.34	1.11
222	2483	2435	48	52	98.07	97.91	4.03		2427	56	130	97.74	94.92	7.49
223	2605	2596	9	7	99.65	99.73	0.61		2570	35	65	98.66	97.53	3.84
228	2053	2013	40	60	98.05	97.11	4.87		1968	85	258	95.86	88.41	16.71
230	2256	2255	1	1	99.96	99.96	0.09		2242	14	14	99.38	99.38	1.24
231	1571	1570	1	1	99.94	99.94	0.13		1560	11	12	99.3	99.24	1.46
232	1780	1152	628	636	64.72	64.43	71.01		1425	355	421	80.06	77.19	43.6
233	3079	3017	62	57	97.99	98.15	3.86		3039	40	41	98.7	98.67	2.63
234	2753	2750	3	3	99.89	99.89	0.22		2749	4	4	99.85	99.85	0.29
TOTAL	109494	105118	4376	4469	96	95.92	8.08		105625	3869	9068	96.47	92.09	11.82

**Table A3:** *DER* Results for individual MIT-BIH AD records, and Total, for *DTT* = 31 samples (86.12 ms), no added noise, and added noise for *SNR* = 6.02 dB.

		DTT = 31 Samples (86.12 ms) No Added Noise							DTT = 31 Samples (86.12 ms) Added Noise for SNR = 6.02 dB					
MIT-BIH AD Record	TB	TP	FN	FP	Se [%]	PPV [%]	DER [%]		TP	FN	FP	Se [%]	PPV [%]	DER [%]
100	2273	2147	126	125	94.46	94.5	11.04		2160	113	114	95.03	94.99	9.99
101	1865	1408	457	460	75.5	75.37	49.17		1517	348	355	81.34	81.04	37.69
102	2187	766	1421	1421	35.03	35.03	129.95		1445	742	806	66.07	64.19	70.78
103	2084	1344	740	737	64.49	64.58	70.87		1593	491	487	76.44	76.59	46.93
104	2229	1639	590	615	73.53	72.72	54.06		1965	264	331	88.16	85.58	26.69
105	2572	2519	53	83	97.94	96.81	5.29		2418	154	247	94.01	90.73	15.59
106	2027	1810	217	217	89.29	89.29	21.41		1830	197	224	90.28	89.09	20.77
107	2137	1107	1030	1026	51.8	51.9	96.21		1707	430	549	79.88	75.66	45.81
108	1763	899	864	1048	50.99	46.17	108.45		1402	361	804	79.52	63.55	66.08
109	2532	613	1919	1916	24.21	24.24	151.46		1439	1093	1098	56.83	56.72	86.53
111	2124	2061	63	63	97.03	97.03	5.93		2054	70	113	96.7	94.79	8.62
112	2539	1510	1029	1029	59.47	59.47	81.06		2079	460	1055	81.88	66.34	59.67
113	1795	693	1102	1103	38.61	38.59	122.84		1170	625	625	65.18	65.18	69.64
114	1879	1836	43	48	97.71	97.45	4.84		1806	73	166	96.11	91.58	12.72
115	1953	397	1556	1556	20.33	20.33	159.34		1094	859	862	56.02	55.93	88.12
116	2412	1523	889	867	63.14	63.72	72.8		2007	405	438	83.21	82.09	34.95
117	1535	1441	94	94	93.88	93.88	12.25		1410	125	1011	91.86	58.24	74.01
118	2278	1068	1210	1210	46.88	46.88	106.23		1501	777	867	65.89	63.39	72.17
119	1987	1936	51	52	97.43	97.38	5.18		1854	133	447	93.31	80.57	29.19
121	1863	1619	244	244	86.9	86.9	26.19		1525	338	1404	81.86	52.07	93.51
122	2476	2471	5	5	99.8	99.8	0.4		2297	179	452	92.77	83.56	25.48
123	1518	317	1201	1198	20.88	20.92	158.04		987	531	580	65.02	62.99	73.19
124	1619	884	735	736	54.6	54.57	90.86		1228	391	644	75.85	65.6	63.93
200	2601	1885	716	717	72.47	72.44	55.09		2167	434	441	83.31	83.09	33.64
201	1963	1078	885	835	54.92	56.35	87.62		1400	563	519	71.32	72.95	55.12
202	2136	960	1176	1172	44.94	45.03	109.93		1420	716	716	66.48	66.48	67.04
203	2980	2047	933	895	68.69	69.58	61.34		2443	537	577	81.98	80.89	37.38
205	2656	2094	562	557	78.84	78.99	42.13		2325	331	335	87.54	87.41	25.08
207	1860	1123	737	742	60.38	60.21	79.52		1499	361	568	80.59	72.52	49.95
208	2955	2126	829	807	71.95	72.49	55.36		2369	586	571	80.17	80.58	39.15
209	3005	1731	1274	1274	57.6	57.6	84.79		2239	766	768	74.51	74.46	51.05
210	2650	1286	1364	1342	48.53	48.93	102.11		1881	769	749	70.98	71.52	57.28
212	2748	2309	439	439	84.02	84.02	31.95		2437	311	312	88.68	88.65	22.67
213	3251	2103	1148	1147	64.69	64.71	70.59		2774	477	477	85.33	85.33	29.34
214	2262	2139	123	119	94.56	94.73	10.7		2159	103	110	95.45	95.15	9.42
215	3363	1612	1751	1750	47.93	47.95	104.1		2283	1080	1079	67.89	67.91	64.2
217	2208	1431	777	777	64.81	64.81	70.38		1842	366	443	83.42	80.61	36.64
219	2154	787	1367	1368	36.54	36.52	126.97		1611	543	604	74.79	72.73	53.25
220	2048	1165	883	883	56.88	56.88	86.23		1593	455	458	77.78	77.67	44.58
221	2427	1125	1302	1296	46.35	46.47	107.05		1754	673	678	72.27	72.12	55.67
222	2483	2301	182	186	92.67	92.52	14.82		2349	134	208	94.6	91.87	13.77
223	2605	1956	649	647	75.09	75.14	49.75		2165	440	470	83.11	82.16	34.93
228	2053	1973	80	100	96.1	95.18	8.77		1931	122	295	94.06	86.75	20.31
230	2256	1390	866	866	61.61	61.61	76.77		1590	666	666	70.48	70.48	59.04
231	1571	1153	418	418	73.39	73.39	53.21		1229	342	343	78.23	78.18	43.6
232	1780	786	994	1002	44.16	43.96	112.13		1154	626	692	64.83	62.51	74.04
233	3079	2182	897	892	70.87	70.98	58.1		2574	505	506	83.6	83.57	32.84
234	2753	1990	763	763	72.28	72.28	55.43		2331	422	422	84.67	84.67	30.66
TOTAL	109494	72740	36754	36847	66.43	66.38	67.22		88007	21487	26686	80.38	76.73	44.00

**Table A4:** *DER* Results for individual MIT-BIH AD records, and Total, for *DTT* = 3 samples (8.33 ms), no added noise, and added noise for *SNR* = 6.02 dB.

		DTT = 3 Samples (8.33 ms) No Added Noise							DTT = 3 Samples (8.33 ms) Added Noise for SNR = 6.02 dB					
MIT-BIH AD Record	TB	TP	FN	FP	Se [%]	PPV [%]	DER [%]		TP	FN	FP	Se [%]	PPV [%]	DER [%]
100	2273	376	1897	1896	16.54	16.55	166.87		562	1711	1712	24.73	24.71	150.59
101	1865	305	1560	1563	16.35	16.33	167.45		461	1404	1411	24.72	24.63	150.94
102	2187	134	2053	2053	6.13	6.13	187.75		429	1758	1822	19.62	19.06	163.69
103	2084	198	1886	1883	9.5	9.51	180.85		344	1740	1736	16.51	16.54	166.79
104	2229	142	2087	2112	6.37	6.3	188.38		367	1862	1929	16.46	15.98	170.08
105	2572	2188	384	414	85.07	84.09	31.03		1949	623	716	75.78	73.13	52.06
106	2027	401	1626	1626	19.78	19.78	160.43		459	1568	1595	22.64	22.35	156.04
107	2137	407	1730	1726	19.05	19.08	161.72		983	1154	1273	46	43.57	113.57
108	1763	99	1664	1848	5.62	5.08	199.21		223	1540	1983	12.65	10.11	199.83
109	2532	19	2513	2510	0.75	0.75	198.38		62	2470	2475	2.45	2.44	195.3
111	2124	123	2001	2001	5.79	5.79	188.42		289	1835	1878	13.61	13.34	174.81
112	2539	7	2532	2532	0.28	0.28	199.45		155	2384	2979	6.1	4.95	211.22
113	1795	199	1596	1597	11.09	11.08	177.88		318	1477	1477	17.72	17.72	164.57
114	1879	74	1805	1810	3.94	3.93	192.39		155	1724	1817	8.25	7.86	188.45
115	1953	4	1949	1949	0.2	0.2	199.59		113	1840	1843	5.79	5.78	188.58
116	2412	64	2348	2326	2.65	2.68	193.78		343	2069	2102	14.22	14.03	172.93
117	1535	142	1393	1393	9.25	9.25	181.5		292	1243	2129	19.02	12.06	219.67
118	2278	4	2274	2274	0.18	0.18	199.65		31	2247	2337	1.36	1.31	201.23
119	1987	793	1194	1195	39.91	39.89	120.23		864	1123	1437	43.48	37.55	128.84
121	1863	664	1199	1199	35.64	35.64	128.72		334	1529	2595	17.93	11.4	221.36
122	2476	2123	353	353	85.74	85.74	28.51		1603	873	1146	64.74	58.31	81.54
123	1518	7	1511	1508	0.46	0.46	198.88		100	1418	1467	6.59	6.38	190.05
124	1619	262	1357	1358	16.18	16.17	167.7		479	1140	1393	29.59	25.59	156.45
200	2601	173	2428	2429	6.65	6.65	186.74		320	2281	2288	12.3	12.27	175.66
201	1963	30	1933	1883	1.53	1.57	194.4		161	1802	1758	8.2	8.39	181.36
202	2136	12	2124	2120	0.56	0.56	198.69		72	2064	2064	3.37	3.37	193.26
203	2980	164	2816	2778	5.5	5.57	187.72		298	2682	2722	10	9.87	181.34
205	2656	574	2082	2077	21.61	21.65	156.59		791	1865	1869	29.78	29.74	140.59
207	1860	147	1713	1718	7.9	7.88	184.46		491	1369	1576	26.4	23.75	158.33
208	2955	342	2613	2591	11.57	11.66	176.11		489	2466	2451	16.55	16.63	166.4
209	3005	83	2922	2922	2.76	2.76	194.48		244	2761	2763	8.12	8.11	183.83
210	2650	64	2586	2564	2.42	2.44	194.34		367	2283	2263	13.85	13.95	171.55
212	2748	851	1897	1897	30.97	30.97	138.06		1128	1620	1621	41.05	41.03	117.94
213	3251	895	2356	2355	27.53	27.54	144.91		1786	1465	1465	54.94	54.94	90.13
214	2262	43	2219	2215	1.9	1.9	196.02		189	2073	2080	8.36	8.33	183.6
215	3363	18	3345	3344	0.54	0.54	198.9		51	3312	3311	1.52	1.52	196.94
217	2208	28	2180	2180	1.27	1.27	197.46		91	2117	2194	4.12	3.98	195.24
219	2154	76	2078	2079	3.53	3.53	192.99		496	1658	1719	23.03	22.39	156.78
220	2048	8	2040	2040	0.39	0.39	199.22		113	1935	1938	5.52	5.51	189.11
221	2427	45	2382	2376	1.85	1.86	196.04		251	2176	2181	10.34	10.32	179.52
222	2483	622	1861	1865	25.05	25.01	150.06		708	1775	1849	28.51	27.69	145.95
223	2605	16	2589	2587	0.61	0.61	198.69		103	2502	2532	3.95	3.91	193.24
228	2053	385	1668	1688	18.75	18.57	163.47		624	1429	1602	30.39	28.03	147.64
230	2256	0	2256	2256	0	0	200		3	2253	2253	0.13	0.13	199.73
231	1571	365	1206	1206	23.23	23.23	153.53		420	1151	1152	26.73	26.72	146.59
232	1780	12	1768	1776	0.67	0.67	199.1		51	1729	1795	2.87	2.76	197.98
233	3079	234	2845	2840	7.6	7.61	184.64		552	2527	2528	17.93	17.92	164.18
234	2753	708	2045	2045	25.72	25.72	148.57		919	1834	1834	33.38	33.38	133.24
TOTAL	109494	14630	94864	94957	13.36	13.35	173.36		21633	87861	93060	19.76	18.86	165.23
